# Complete Genome Sequence of Aggregatibacter actinomycetemcomitans Strain CU1000N

**DOI:** 10.1128/mra.01042-21

**Published:** 2022-03-07

**Authors:** Carla Cugini, Karthikeyan Nattarayan, Rachel L. Ehrlich, Azad Ahmed, Narayanan Ramasubbu

**Affiliations:** a Department of Oral Biology, Rutgers School of Dental Medicine, Newark, New Jersey, USA; b Center for Genomic Sciences, Institute for Molecular Medicine and Infectious Disease, Department of Microbiology and Immunology, Drexel University College of Medicine, Philadelphia, Pennsylvania, USA; c Genomics Core Facility, Clinical and Translational Research Institute, Drexel University College of Medicine, Philadelphia, Pennsylvania, USA; University of Arizona

## Abstract

Here, we report the complete genome sequence of Aggregatibacter actinomycetemcomitans strain CU1000N. This rough strain is used extensively as a model organism to characterize localized aggressive periodontitis pathogenesis, the basic biology and oral cavity colonization of A. actinomycetemcomitans, and its interactions with other members of the oral microbiome.

## ANNOUNCEMENT

Aggregatibacter actinomycetemcomitans is a Gram-negative, nonmotile, facultative anaerobe of the oral microbiota that is implicated in the development of localized aggressive periodontitis (LAP) (1, 2). A. actinomycetemcomitans strain CU1000 was isolated in 1992 in New York City from the first-molar site of a 13-year-old, medically healthy, African American female patient with classic symptoms of LAP (3). CU1000N is a spontaneous nalidixic acid-resistant (DNA gyrase point mutant) strain of CU1000 (4). Despite the lack of a complete genome sequence, CU1000N, a “rough” strain, is commonly used as a model for pathogenesis and basic bacteriology studies (3–15). Rough strains of A. actinomycetemcomitans display the classic star-shaped colony morphology observed in clinical isolates from LAP patients; therefore, they are more appropriate for the study of A. actinomycetemcomitans biology than are those displaying a “smooth” colony phenotype (16, 17).

The CU1000N isolate was kept as a frozen stock at −80°C and was two passages from the original stock of the spontaneous mutant. It was streaked for isolation from −80°C to an entire Trypticase soy broth agar plate supplemented with glucose (8 g/L), sodium bicarbonate (10% [wt/vol]), and yeast extract (6 g/L) and was grown for 2 days under microaerophilic conditions (10% carbon dioxide). The rough phenotype was confirmed microscopically from the single colonies on the plate. Cells were scraped from the agar plate from the primary streak, resuspended in 2 mL of AE buffer (Qiagen), centrifuged (16,000 × *g* at 4°C for 2 min), homogenized, and resuspended in 100 μL of AE buffer. The genomic DNA was purified by phenol-chloroform-ethanol precipitation (18). DNA integrity was verified by agarose gel electrophoresis, purity was evaluated spectrophotometrically, and the concentration was determined fluorometrically.

Libraries were created for sequencing using the multiplex microbial SMRTbell library preparation protocol for the PacBio Sequel system. The SMRTbell template preparation kit 1.0-SPv3 and a SMRTbell barcoded adapter 96-well plate were used. SMRTbell libraries were pooled, size selected using BluePippin with a 5,000-bp cutoff value, and sequenced on a PacBio Sequel system. The polymerase reads were demultiplexed and broken into subreads (PacBio single-molecule real-time [SMRT] Link v7.0.1). The sequencing produced 47,445 polymerase reads and 47,057 postfiltered subreads (*N*_50_, 9,973 nucleotides [nt]). Filtering as an intermediate step in assembling the reads and *de novo* assembly of the subreads were performed using Falcon (PacBio SMRT Link v7.0.1), which yielded a single contig of 2,337,866 nt (19). The resulting contig was circularized and permuted to *dnaA* using Circlator v1.5.5 (20). Errors were corrected using Arrow (PacBio SMRT Link v7.0.1). The final assembly was 2,331,529 nt and had an average coverage of 703×. It had a GC content of 44.2%, consistent with other completed A. actinomycetemcomitans genomes. The species of the final assembly was verified using taxator-tk v1.2 with the nonredundant-microbial_20140513 database (refpack from http://research.bifo.helmholtz-hzi.de/software) (21).

Annotation was performed by NCBI using the Prokaryotic Genome Annotation Pipeline (PGAP) v5.2 (22). The chromosome contains 2,268 genes, including 2,191 coding sequences, 19 rRNAs, 54 tRNAs including selenocysteine, 3 noncoding RNAs, and 1 predicted CRISPR.

### Data availability.

This whole-genome project has been deposited in DDBJ/ENA/GenBank under the accession number CP076449. The raw reads have been deposited in the NCBI SRA under the BioProject accession number PRJNA735719. The version described in this paper is the first version.
